# Connecting Female Entertainment Workers in Cambodia to Health Care Services Using mHealth: Economic Evaluation of Mobile Link

**DOI:** 10.2196/52734

**Published:** 2024-07-25

**Authors:** Anton L V Avanceña, Carinne Brody, Pheak Chhoun, Sovannary Tuot, Siyan Yi

**Affiliations:** 1 Health Outcomes Division College of Pharmacy The University of Texas at Austin Austin, TX United States; 2 Department of Internal Medicine Dell Medical School The University of Texas at Austin Austin, TX United States; 3 Public Health Program College of Education and Health Sciences Touro University California Vallejo, CA United States; 4 KHANA Center for Population Health Research Phnom Penh Cambodia; 5 Faculty of Social Sciences and Humanity Royal University of Phnom Penh Phnom Pehn Cambodia; 6 Department of Community and Global Health Graduate School of Medicine The University of Tokyo Tokyo Japan; 7 Saw Swee Hock School of Public Health National University of Singapore Singapore Singapore

**Keywords:** female entertainment workers, Cambodia, mHealth, mobile health, economic evaluation, stigmatized populations, women's health, sexual health, STI, sexually transmitted infection, STD, sexually transmitted disease, economic, cost, costs, affordable, affordability, budget, finance, financial

## Abstract

**Background:**

Mobile Link is a mobile phone–based intervention to increase access to, and use of, health care services among female entertainment workers in Cambodia who face higher risks for specific diseases and gender-based violence. A multisite randomized controlled trial showed that Mobile Link connected female entertainment workers with outreach workers for information and escorted referrals after 6 months but did not lead to statistically significant improvements in HIV and sexually transmitted infection testing, contraceptive use, and condom use.

**Objective:**

This study aims to conduct a 3-part economic evaluation of Mobile Link to understand its costs, value, and affordability.

**Methods:**

We conducted cost, cost-effectiveness, and budget impact analyses of Mobile Link using cost and outcomes data from the Mobile Link trial and other sources. For the cost analysis, we estimated the total, per-person, and incremental costs of Mobile Link compared with usual care. Using probabilistic decision-analytic models, we estimated the 1-year cost-effectiveness of Mobile Link from payer and combined payer and patient perspectives by converting selected primary and secondary outcomes from the trial to disability-adjusted life years (DALYs) averted. Finally, we estimated the financial costs of scaling up Mobile Link’s messaging and outreach services to 70% of female entertainment workers in 5 years.

**Results:**

The incremental costs of Mobile Link were US $199 from a payer perspective and US $195 per person from a combined payer and patient perspective. With an average of 0.018 (95% predicted interval –0.088 to 0.126) DALYs averted, Mobile Link’s cost-effectiveness was US $10,955 per DALY from a payer perspective (US $10,755 per DALY averted from a payer and patient perspective). The costs of Mobile Link would have to decrease by 85%, or its effectiveness would have to be 5.56 times higher, for the intervention to meet the upper limit of recommended cost-effectiveness thresholds in Cambodia (US $1671 per DALY averted). The 5-year cost of scaling Mobile Link to 34,790 female entertainment workers was estimated at US $1.64 million or US $46 per person per year.

**Conclusions:**

This study provided a comprehensive economic evaluation of Mobile Link. We found that Mobile Link is not likely to be cost-effective unless its costs decrease or its effectiveness increases. Scaling up Mobile Link to more female entertainment workers is estimated to cost less than the costs of the trial. Given the importance of linking female entertainment workers to essential services, future research should focus on enhancing the effectiveness of Mobile Link or developing new mobile health interventions for this population.

**Trial Registration:**

ClinicalTrials.gov NCT03117842; https://clinicaltrials.gov/study/NCT03117842

## Introduction

Female entertainment workers in Cambodia face many structural barriers to accessing and using health services such as stigma and criminalization of sex work [1]. As a result, despite facing higher risks of HIV and other sexually transmitted infections (STIs), gender-based violence (GBV), and being forced to drink while working, female entertainment workers infrequently use health services and can be hard to reach by health care workers [2]. To improve the health of female entertainment workers, achieve broader public health goals (eg, reduction of HIV burden), and advance health equity, new tools that effectively and efficiently engage female entertainment workers in health services are critical and necessary.

Mobile phone–based health interventions, often referred to as mobile health (mHealth) interventions, have been developed and tested in several settings globally to better reach stigmatized populations such as female entertainment workers. However, results from those studies have been mixed [3-6]. In Cambodia, an mHealth intervention called Mobile Link was developed, following formative participatory research [7], to engage with and connect female entertainment workers to essential prevention, care, and treatment services using automated SMS text messaging and voice messages. Its effectiveness in reducing risk behaviors and increasing the use of health care services was evaluated in a randomized controlled trial (RCT) conducted from March 2018 to June 2019 at 5 sites [8]. The trial found that Mobile Link helped connect female entertainment workers with outreach workers for information and escorted referrals but did not lead to statistically significant improvements in HIV and STI testing, contraceptive use, and condom use in adjusted models [9].

The adoption and scale-up of Mobile Link and other mHealth interventions will depend on their effectiveness, value, and financial costs [10]. This study conducted a 3-part economic evaluation of Mobile Link to understand its costs, cost-effectiveness, and budget impact. We aim to estimate the costs of Mobile Link, the financial requirements and affordability of a potential scale-up, and the short-term value of the intervention using disability-adjusted life years (DALYs) averted as a measure of health benefit. Findings from this study can be used by decision makers considering the rollout of Mobile Link in other Cambodian jurisdictions, as well as to inform future economic evaluations of other mHealth interventions [11].

## Methods

### Study Design

This study involves 3 distinct empirical and model-based evaluations. The cost analysis determines the total, per-person, and incremental costs of Mobile Link using expenditure data from the trial and other data sources. The model-based cost-effectiveness analysis (CEA) evaluates whether Mobile Link offers “value for money” based on the efficacy results of the trial and the results of the cost analysis. Finally, the budget impact analysis estimates the financial or monetary cost of scaling up Mobile Link to more female entertainment workers in Cambodia. We followed guidelines in the economic evaluation including the ISPOR principles of good practice for budget impact analysis, Consolidated Health Economic Evaluation Reporting Standards, and specific guidance for evaluating mHealth and digital health technologies [10-13]. We used payer and combined payer and patient perspectives in this study. The Impact Inventory [14] (Multimedia Appendix 1) lists the costs and benefits included in each perspective. Patient-level data were analyzed using Stata/SE 15.1 (StataCorp LLC), and economic analyses were done in Microsoft Excel (Microsoft Corp).

### Mobile Link Trial

#### Overview

Details on the Mobile Link RCT are available in a previously published protocol [8] (trial registration NCT03117842 in ClinicalTrials.gov), and the trial results are presented in separate publications [9,15]. In summary, female entertainment workers were recruited by community health workers in Phnom Penh, Battambang, Banteay Meanchey, and Siem Reap and randomly assigned to the treatment and control arms of the trial. Participants in the treatment arm received health information, reminders, and referral resources in Khmer through SMS text messaging or voice messages. Female entertainment workers in the control arm received usual care, which included in-person counseling, HIV and STI testing, condoms, and access to a toll-free hotline with trained counselors. Health messages were developed after a series of formative participatory research and a review of behavior change theories [7]. Themes covered in messages included cervical cancer; contraception; HIV and STI prevention; miscarriage, pregnancy, and pregnancy termination; alcohol use at work; vaginal health and hygiene; GBV; and general health information. Messages were delivered twice a week for 10 weeks, and messages about each theme were repeated every 10 weeks for 60 weeks. Each health message was followed by another message giving female entertainment workers the option to be linked to an outreach worker. Participants who chose this option were contacted by a Mobile Link staff who provided individualized information via phone or in person and escorted participants to services when requested.

After losing 730 participants in the follow-up, the final analytic sample included 388 participants: 218 in the treatment group and 170 in the control group (Multimedia Appendix 2). The dropout rate is notable although not unexpected in research involving hard-to-reach and stigmatized populations [16]. Statistical analyses found no significant differences between the participants who dropped out of the study and those who stayed in the trial. In crude tests of association, statistically significant differences by province and entertainment venue were identified between the treatment and control groups.

#### Outcomes and Measures

The primary outcome measures were (1) HIV testing, (2) STI testing when experiencing symptoms, (3) modern contraceptive use, (4) condom use with nonpaying partners, and (5) condom use with paying partners. Secondary outcome measures were (1) contact with outreach workers; (2) use of referrals while being escorted by outreach workers (ie, escorted referrals); (3) forced drinking at work; and (4) experience with GBV (full descriptions of these outcomes are available in the trial protocol and report [8,9]). Outcomes were measured using self-reported data collected using questionnaires administered via a tablet. Data were collected at 3 time points: baseline, midline (6 months after the baseline), and endline (12 months after the baseline).

Multimedia Appendices 3 and 4, respectively, show the primary and secondary outcomes from the trial analyzed per protocol. No statistically significant differences between intervention and control groups were observed for any primary outcome in fully adjusted multilevel logistic regression models with mixed effects controlling for venue type, province, cohabitation, age, education, and outreach worker contact [9]. Among the secondary outcomes (Multimedia Appendix 4), contact with an outreach worker in the last 6 months, escorted referrals in the last 6 months, and no forced drinking at work in the last 3 months were all found to be associated with the intervention in crude analyses. After adjusting for venue type, province, cohabitation, age, and education in logistic regression, only 2 secondary outcomes—escorted referral and forced drinking at work—were associated with Mobile Link.

### Cost Analysis

Using a top-down or gross approach [10,17], we estimated costs from the perspective of payers and patients. Payer costs, including startup (ie, fixed) costs and other variable costs, were estimated using expenditure data collected during the trial (Table 1). The Global Fund to Fight AIDS, Tuberculosis, and Malaria (Global Fund), which supports several programs for female entertainment workers in Cambodia, assumed most of the costs including activities and services associated with usual care available to participants in the control and treatment groups. Additional costs associated with the implementation of the Mobile Link intervention were borne by L’Initiative/Expertise France, the trial funder. We evenly divided the Global Fund costs among all participants in the control and intervention groups, while Mobile Link costs were apportioned to participants in the treatment group only.

**Table 1:** Payer costs (in US $) from Mobile Link trial. Costs have been roughly classified by activity (diagnosis and treatment; information, education, and communication; and program management) and type of input (consumables and personnel). Global Fund costs were spent on both control and intervention groups, while Expertise France costs were only spent on the intervention arm.

Cost category		L’Initiative/Expertise France (US $)	Global Fund (US $)
**Diagnosis and treatment**			
	Consumables	N/A^a^	70,871
	Personnel	N/A	3800
	Subtotal	N/A	74,671
**Information, education, and communication**			
	Consumables	125,767	64,754
	Personnel	34,314	254,433
	Subtotal	160,081	319,186
**Program management**			
	Consumables	N/A	2500
	Personnel	3099	N/A
	Subtotal	3099	2500
Total		163,180	396,357

^a^N/A: not applicable.

The primary patient cost we considered was time costs associated with seeking health services [18], which were reduced by contacting an outreach worker. Based on interviews with trial participants, the average time to access health services after contact with an outreach worker was 36 minutes (0.6 h); without outreach workers, the time to access health services was 2.2 hours. Therefore, the net time benefit to female entertainment workers was 1.6 hours per contact with an outreach worker. Based on baseline data from the trial [15], we assumed that participants engaged with female entertainment workers at least twice during the trial, for a total of 3.2 hours in net time benefits.

Following best practices [18], we used earnings data among female entertainment workers to value their time costs. With average weekly earnings of US $270 (Multimedia Appendix 1) and assuming an average of 40 work hours per week, the estimated savings from care-seeking after contact with an outreach worker is US $21.60 (US $6.75 earnings per hour × 3.2 hours). We multiplied this cost by the proportion of female entertainment workers who contacted an outreach worker in each trial arm to get the total patient cost savings and then subtracted the result from the total payer costs. Since basic health care services, such as sexual and reproductive health services, are available for free to female entertainment workers through government- and grant-funded programs, we did not include out-of-pocket spending. Due to data limitations, we excluded transportation costs and other costs associated with particular illnesses such as STIs.

We present total costs, cost per participant, and incremental costs between usual care and the Mobile Link intervention. All costs are reported in 2019 US $, the year the RCT concluded.

### CEA Methods

#### Overview

**Figure 1:**
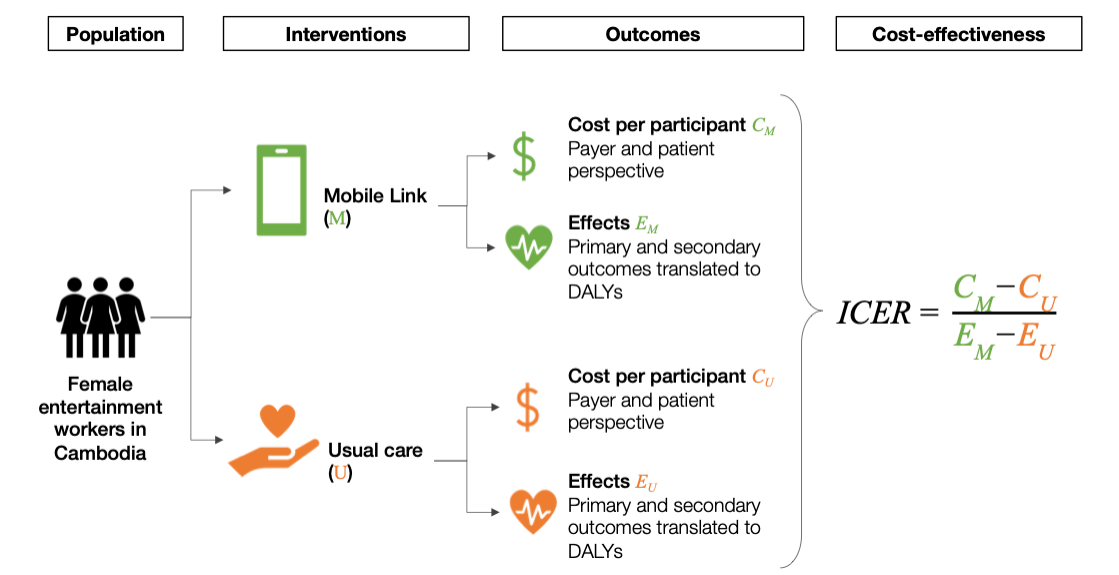
CEA overview. This graphic provides an overview of the analytical approach used in the CEA. Costs and benefits were estimated and valued for Mobile Link and usual care separately using data from the trial and other sources. The ICER was then calculated by dividing the incremental costs by the incremental benefit. C: cost; CEA: cost-effectiveness analysis; DALY: disability-adjusted life year; E: effect; ICER: incremental cost-effectiveness ratio; M: Mobile Link; U: usual care.

An overview of the CEA is shown in Figure 1. Costs (*C*_M_ and *C*_U_ in Figure 1) were based on the cost analysis described previously. The health effects of Mobile Link (*E*_M_ and *E*_U_ in Figure 1) were estimated using the efficacy results from the trial, which we translated into DALYs averted. Because the Mobile Link trial found no statistically significant differences in primary outcomes between the control and intervention groups, we used a probabilistic model that incorporates the uncertainty around the treatment effects. Several factors, such as sample size and the types or anticipated incidence of outcomes, affect whether studies are sufficiently powered to estimate treatment effects [19,20]. In the Mobile Link trial specifically, the high (784/1118, 65%) dropout rate among female entertainment workers may have affected the results. Thus, null results from hypothesis testing do not automatically denote zero treatment effects [20,21]. Our probabilistic economic evaluation is designed to determine the probability that Mobile Link is cost-effective compared with usual care, given the uncertainty in the available evidence [22-24].

#### Estimating Health Effects

We included 5 outcomes from the trial in the CEA: HIV testing, STI testing, modern contraceptive use, forced drinking at work, and experience with GBV. These outcomes were selected because they could be translated into DALYs, a commonly used measure of health and disease in low- and middle-income countries (Multimedia Appendix 5 [1,25-41]) [42]. DALYs measure mortality and morbidity in 1 metric and are calculated by adding years of life lost and years of life with disability. Because the time horizon of the analysis is 1 year, no years of life lost were included, and the DALYs associated with each outcome were equal to the disability weights used to estimate years of life with disability.

Disability weights range from 0 (perfect quality of life) to 1 (disease burden equivalent to being dead). We took disability weights from the 2019 Global Burden of Diseases study [25] and the Marie Stopes International Impact 2 Model (version 5) [26,27], a simulation model that estimates the effect of contraceptive use on maternal and child health outcomes (Table 2). For HIV and STI testing, we made further adjustments to the disability weights to reflect the prevalence of the disease and the probability of being treated after a positive test (full details are in Multimedia Appendix 6). For the probabilistic analysis, we assigned β distributions to each disability weight and used the mean values and SDs to estimate the α and β parameters.

**Table 2:** Disability weights assigned to the Mobile Link outcomes.

Outcome of Mobile Link trial	Description	Disability weight, mean (SD)	Source
HIV testing	Disability associated with undiagnosed and untreated early HIV infection	0.147 (0.085)	GBD^a^ 2019
STI^b^ testing	Disability associated with a mild and acute STI episode	0.006 (0.002)	GBD 2019
Modern contraceptive use	Maternal disability associated with nonuse of modern contraceptives	0.014 (0.008)	Impact 2
Forced drinking at work	Disability associated with very mild alcohol use disorder	0.123 (0.063)	GBD 2019
Gender-based violence	Disability associated with physical and mental harms and injuries	0.211 (0.109)	GBD 2019

^a^GBD: Global Burden of Diseases study.

^b^STI: sexually transmitted infection.

To estimate the total DALYs averted by Mobile Link, we summed the product of the absolute risk difference (ARD) and the DALYs for each of the 5 outcomes, represented by the index *i* in Equation 1. When interpreting and applying nonsignificant results, the ARD (also called absolute risk reduction) is recommended to communicate the potential magnitude of the effect of an intervention [43]. We calculated ARDs by subtracting the risk (or probability) of each outcome in the usual care group (*R*_U_)from the risk of each outcome in the Mobile Link group (*R*_M_) at endline (Table 3) [44]. It is worth noting that for HIV testing and modern contraceptive use, Mobile Link is associated with a negative ARD, which means it performs worse on these outcomes than usual care on average (Table 3).



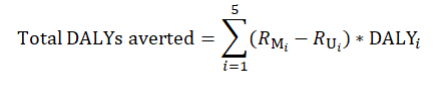



**Table 3:** Absolute risk differences for primary and secondary outcomes. Only participants with data at the midline and endline periods were included in calculating risks. Means and SDs in parentheses are provided.

Outcome			Risk in control group (%), mean (SD)^a^		Risk in intervention group (%), mean (SD)		Absolute risk difference in percentage points
**Primary outcomes**							
	Tested for HIV, last 6 months	70.25 (5.93)		68.93 (5.82)		–1.32^b^	
	Tested for STIs^c^, most recent symptoms	29.17 (2.46)		39.1 (3.3)		9.93	
	Use of modern contraceptives to prevent pregnancy	41.47 (3.5)		40.05 (3.38)		–1.42^b^	
**Secondary outcomes**							
	No forced drinking at work, last 3 months	66.36 (5.6)		67.85 (5.73)		1.49	
	Low or no gender-based violence	40.09 (3.39)		48.23 (4.07)		8.14	

^a^The incidence of the outcome in the control or unexposed group is also called baseline risk.

^b^In the base case, Mobile Link performed more poorly than the standard of care for these outcomes, though the differences were not statistically significant.

^c^STI: sexually transmitted infection.

To characterize the uncertainty in ARDs, we assigned normal (base-case assumption), β, and uniform distributions to each group’s endline risk and generated 10,000 estimates of the ARD for each outcome. Following recommended procedures [43,45], we derived the lower and upper values of each risk using the confidence intervals of odds ratios reported in the trial (Multimedia Appendices 2 and 3). We assumed that the risks reported in the trial were the mean values, and the SD was equal to the difference between the mean value and upper limit divided by 1.96.

### Incremental and Sensitivity Analyses

We summarized the cost-effectiveness results in incremental cost-effectiveness ratios (ICERs). The ICER is calculated by dividing the net costs of intervention by its net effectiveness (Figure 1) and represents the cost of each DALY averted. ICERs expressed in cost per DALY averted can be compared with context-specific cost-effectiveness threshold to determine whether an intervention is efficient. From a supply-side perspective, the threshold is a measure of opportunity cost or the amount of health displaced by additional spending in the health sector [46]. Alternatively, from a demand-side perspective, the threshold represents a decision maker’s willingness to pay for an additional unit of benefit. While a cost-effectiveness threshold has not been empirically measured for Cambodia, several ranges based on the country’s per-capita gross domestic product have been proposed [47-49]. This study used 50% to 100% of Cambodia’s 2019 per-capita gross domestic product as the cost-effectiveness threshold range (US $835-US $1671 per DALY averted) [50].

We calculated average ICERs across 10,000 model simulations and the associated 95% predicted interval, which represents the 5th and 95th percentiles of the simulated results. We also constructed a cost-effectiveness acceptability frontier, which plots the intervention that is most likely to be cost-effective over a range of cost-effectiveness thresholds.

### Budget Impact Analysis

We conducted a budget impact analysis to estimate the undiscounted financial cost and affordability of delivering Mobile Link to female entertainment workers in Cambodia [13]. We focused on the costs of scaling up Mobile Link’s messaging and outreach services and excluded the costs of health care services used by female entertainment workers or any potential long-term savings from improved health care service use among female entertainment workers. We assumed a 5-year time horizon where an additional 14% (n=6958) of the 50,000 estimated female entertainment workers in Cambodia [51] are provided access to Mobile Link annually, culminating in a 70% overall coverage rate. The analysis was conducted from a payer perspective.

We used capital costs (eg, mobile platform development and beta testing) from Table 1 in the budget impact analysis. We only applied these fixed costs in the first year since they can be leveraged to scale up Mobile Link across Cambodia without additional investments in the short- to medium-term. We also used 2 variable costs to reflect the expected economies of scale with expanding Mobile Link. The cost of providing messaging services to each additional female entertainment worker was estimated to be US $2.66, which included all costs associated with weekly SMS text messaging to female entertainment workers (Mobile Link trial team and personal communication). Assuming 280 female entertainment workers per outreach worker, we assumed a per-person annual cost of US $9.54 for outreach workers, which included salaries and costs for training, communication, and travel.

### Ethical Considerations

This study used secondary data and did not involve human participants; therefore, it is exempt from ethical review. The Mobile Link trial was approved by the National Ethics Committee for Health Research (NECHR; 142NECHR) of the Ministry of Health in Cambodia and the Touro College Institutional Review Board (PH-0117).

## Results

### Cost Analysis Results

The cost of the Mobile Link intervention from a payer perspective was US $352,382 or US $429 per person (Multimedia Appendix 7). After subtracting the costs of usual care (US $207,155 or US $230 per person), the incremental cost of Mobile Link was US $145,226 or US $199 per person. From a combined payer and patient perspective, the incremental cost per person of Mobile Link was reduced to US $195.

### CEA Results

Table 4 presents the average DALYs averted from the probabilistic models. A total of 2 outcomes—HIV testing and modern contraceptive use—were associated with negative mean DALYs averted, which implies that usual care produced with fewer DALYs than Mobile Link across 10,000 simulations. The remaining 3 outcomes—STI testing, modern contraceptive use, and forced drinking at work—were associated with positive mean DALYs averted, although 95% predicted intervals in all 3 models included negative values.

**Table 4:** Average DALYs^a^ averted by the outcome and assumed distribution of risks. DALYs averted were calculated by comparing Mobile Link with usual care. Means and 95% predicted intervals in parentheses from 10,000 model simulations are presented under different assumptions around the distribution of endline risks among control and intervention groups (normal, β, and uniform).

Outcome	Mean DALYs averted, 95% predicted interval		
	Normal distribution^b^	β distribution	Uniform distribution
HIV testing	–0.002 (–0.022 to 0.019)	–0.002 (–0.023 to 0.018)	–0.0004 (–0.042 to 0.04)
STI^c^ testing	0.0006 (–0.0014 to 0.0027)	0.0006 (–0.0014 to 0.0027)	0.0003 (–0.0021 to 0.0027)
Modern contraceptive use	–0.0002 (–0.0024 to 0.0019)	–0.0002 (–0.0024 to 0.002)	–0.0001 (–0.004 to 0.0039)
Forced drinking at work	0.002 (–0.018 to 0.022)	0.002 (–0.018 to 0.022)	0.0004 (–0.042 to 0.043)
Gender-based violence	0.017 (–0.084 to 0.121)	0.018 (–0.087 to 0.118)	0.003 (–0.117 to 0.121)
Total	0.018 (–0.088 to 0.126)	0.018 (–0.092 to 0.122)	0.003 (–0.131 to 0.135)

^a^DALY: disability-adjusted life year.

^b^Base-case assumption.

^c^STI: sexually transmitted infection.

Using the per-person incremental costs (US $199; Multimedia Appendix 7) and the base-case DALYs averted (0.018; Table 4), the ICER of Mobile Link was US $10,955 per DALY averted from a payer perspective and US $10,755 per DALY averted from a payer and patient perspective. These ICERs are more than 600% of the per-capita GDP of Cambodia in 2019.

The model estimated that the incremental cost of Mobile Link would have to be reduced by 85% (from US $199 to US $30 per person) for the ICER of Mobile Link to meet the US $1671 per DALY averted cost-effectiveness threshold without any corresponding changes in its effectiveness. Alternatively, the DALYs averted of Mobile Link would have to be 5.56 times higher (from 0.018 to 0.119) for Mobile Link to meet the same cost-effectiveness threshold without changes in its cost.

**Figure 2:**
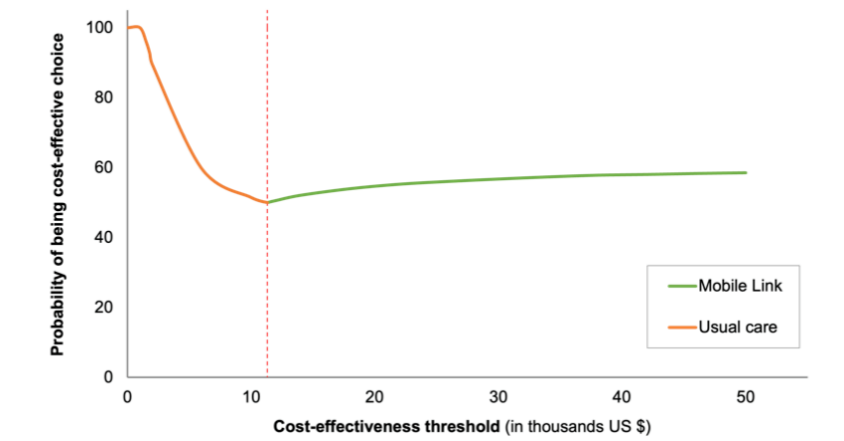
Cost-effectiveness acceptability frontier assuming a normal distribution for endline risks. The red dashed line denotes the cost-effectiveness threshold where the optimal strategy changes.

**Figure 3:**
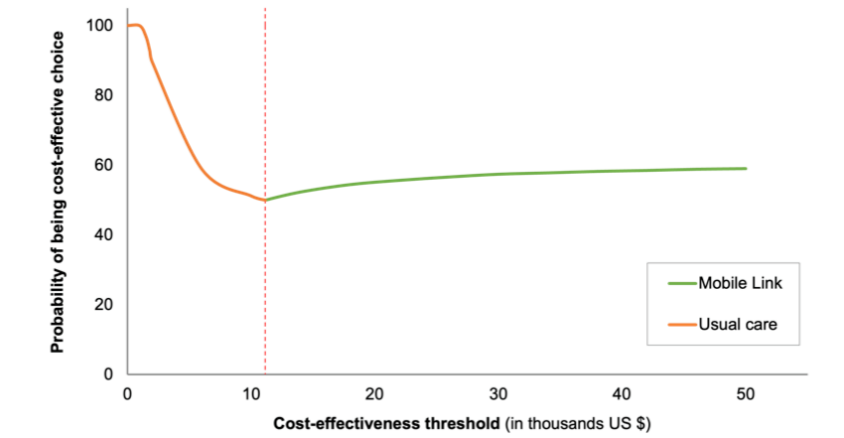
Cost-effectiveness acceptability frontier assuming a β distribution for endline risks. The red dashed line denotes the cost-effectiveness threshold where the optimal strategy changes.

**Figure 4:**
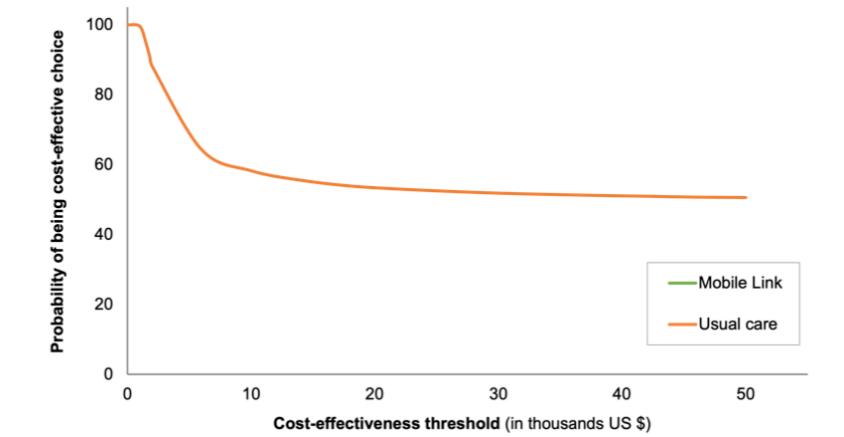
Cost-effectiveness acceptability frontier assuming a uniform distribution for endline risks.

The results of the sensitivity analysis are shown in Figures 2-4. At a threshold of US $1671 per DALY averted, the model found that usual care has a 100% probability of being the cost-effective option. Assuming normal and β distributions for risks, the probability that Mobile Link was the cost-effective intervention (vs usual care) increased as the threshold increased. However, assuming a uniform distribution for risks, usual care was most likely to be the cost-effective intervention event at higher threshold values (ie, ≤US $50,000 per DALY gained).

### Budget Impact Analysis Results

**Figure 5:**
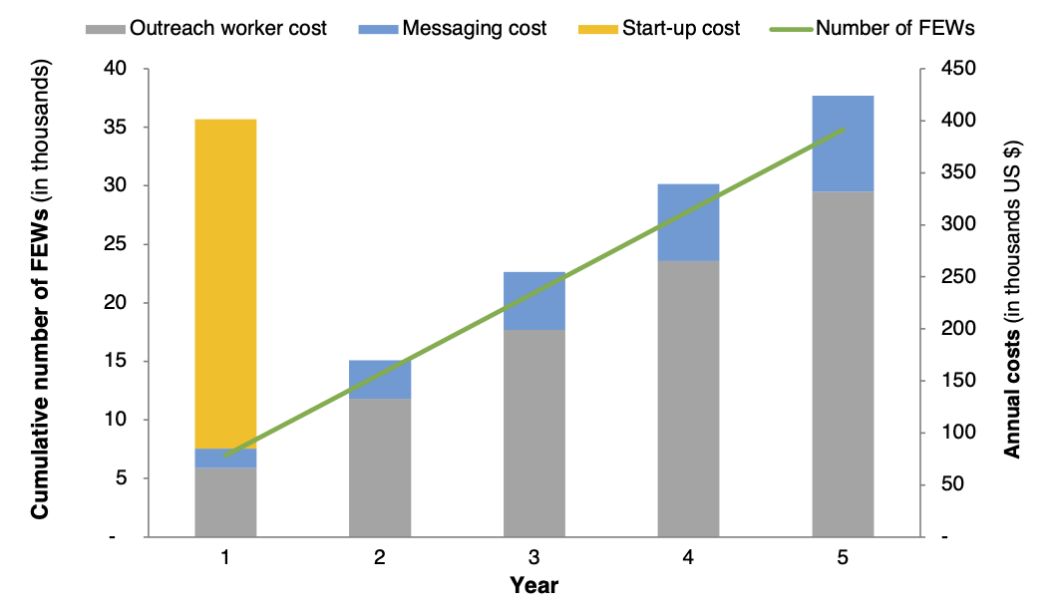
Five-year budget impact of scaling up Mobile Link in Cambodia. This graph has 2 axes; the left axis shows the cumulative number of FEWs receiving the Mobile Link intervention. The right axis plots the total annual cost (in US $) of the scale-up disaggregated by the type of cost (start-up, messaging, and outreach workers). FEW: female entertainment worker.

The results of the budget impact analysis are shown in Figure 5. The 5-year budget impact of scaling up Mobile Link to 70% (34,790/50,000) of female entertainment workers was approximately US $1.59 million. In total, 62% of the total costs were associated with increasing the number of outreach workers, and 17% were from the messaging service. The average annual cost was approximately US $318,000 or US $46 per person.

## Discussion

### Principal Findings

This economic evaluation estimated the costs, cost-effectiveness, and budget impact of Mobile Link, a mHealth intervention designed to increase access to health care services among female entertainment workers in Cambodia. We translated 5 trial outcomes to DALYs averted and found that Mobile Link is likely to avert more DALYs than usual care in our probabilistic model. However, we also found that Mobile Link has a low probability of being a cost-effective intervention in Cambodia.

Using cost data from the trial, we found that the ICER of Mobile Link—from both payer and combined patient and payer perspectives—was higher than the commonly used cost-effectiveness thresholds in Cambodia. Our model suggests that Mobile Link’s costs would need to decrease, or its effectiveness would need to increase, for the intervention to be considered cost-effective. With sufficient economies of scale, Mobile Link may reach the cost thresholds needed to lower its ICER. For example, our budget impact analysis suggests that the financial cost of scaling up Mobile Link is US $46 per female entertainment worker, only 23% of the trial-based cost estimate. This figure is likely overestimated because we did not account for possible cost savings to the health care system. A microcosting study that identifies the essential inputs in scaling up Mobile Link can be conducted to determine the total financial costs outside of a trial setting.

### Limitations

Several limitations in this study must be noted to guide the interpretation of the results. First, our cost analysis and CEA used a 1-year time horizon, and many of Mobile Link’s longer-term benefits may have been underestimated or excluded. For example, STI testing leads to higher rates of treatment, which may reduce STI rates among female entertainment workers and their clients. A follow-up study among the trial participants can evaluate whether Mobile Link has lasting benefits beyond 1 year that should be valued and included in an economic evaluation. Second, the use of trial costs may bias our estimates. Protocol-driven costs often overestimate the actual costs of intervention because significant resources are allocated to ensure adherence to trial design such as blinding, conducting multiple follow-ups, and sampling procedures [22,24]. In practice, economies of scale may reduce the cost of scaling up Mobile Link to larger numbers of female entertainment workers, and we partially addressed this issue by using the expected marginal cost of Mobile Link in the budget impact analysis. Third, many of the trial’s outcomes were intermediate measures of health (eg, STI testing, contact with an outreach worker), which we translated into DALYs averted using commonly used methods. As a result, we may have underestimated the total health effects of Mobile Link, which future studies should explore. Fourth, the trial experienced a high dropout rate from participants, which is often expected when working with stigmatized populations such as female entertainment workers [16]; however, this may have affected the ability of the study to identify statistically significant differences between the control and intervention groups. We addressed this limitation in our CEA by conducting a probabilistic analysis that accounted for the uncertainty in trial outcomes. Additional research efforts that use effective retention strategies can re-evaluate the effect of Mobile Link and other mHealth interventions on care-seeking among female entertainment workers. Finally, we used a limited perspective in our economic evaluations, which may have undervalued the nonhealth benefits of Mobile Link. Future studies may include other health care sectors and societal costs [10].

### Comparison With Prior Work

Prior economic evaluations have shown that mHealth interventions can be cost-effective in various low-resource settings [52,53]. For example, a CEA of a mHealth intervention designed to increase postabortion family planning in Cambodia found that it had a high probability of being cost-effective when the highest thresholds were used [28]. A recent systematic review also found that mHealth interventions for pregnant women are low-cost or cost-effective [54]. In India, a few mHealth interventions for maternal, child, and infant health have been shown to provide high value [55-57].

The high ICERs reported in this study may reflect several factors including our use of trial costs and the difficulties associated with reaching and serving a highly stigmatized population such as female entertainment workers in Cambodia. The criminalization of sex work has driven female entertainment workers into riskier environments and arrangements, and the informal nature of their work prevents them from organizing and demanding better work conditions [1,8,29]. Without changes to the structural barriers to care, interventions targeted to female entertainment workers may continue to be costlier and less effective than interventions for less stigmatized populations.

Finally, Mobile Link may still offer good value if it achieves other health care goals such as equal access to services and equity [58]. Previous qualitative research on Mobile Link has demonstrated that female entertainment workers appreciate the convenience, benefits, and resources offered by the intervention [59]. These aspects are difficult to include in an economic evaluation but should be included in deliberative decision-making.

### Conclusions

This economic evaluation provided estimates of the cost, cost-effectiveness, and budget impact of Mobile Link, an mHealth intervention that engages and connects female entertainment workers in Cambodia to essential health care services. Using cost and outcomes data from the Mobile Link trial and other sources, this study found that Mobile Link may improve the health and health care access of female entertainment workers in Cambodia, but it is unlikely to offer sufficient economic value. Given the importance of linking female entertainment workers to essential services, future research should focus on enhancing the effectiveness of Mobile Link or developing new mHealth interventions for this population.

## Appendix

Multimedia Appendix 1 Impact Inventory.

Multimedia Appendix 2 Baseline characteristics of the final analytical sample (n=388) in the Mobile Link trial.

Multimedia Appendix 3 Primary outcomes among the analytic sample from Mobile Link randomized controlled trial.

Multimedia Appendix 4 Secondary outcomes among the analytic sample from Mobile Link randomized controlled trial.

Multimedia Appendix 5 Calculating disability weights for each Mobile Link trial outcome.

Multimedia Appendix 6 Disability weights from the Global Burden of Diseases study used in the cost-effectiveness analysis.

Multimedia Appendix 7 Total cost of Mobile Link and average cost per participant from payer and combined payer and patient perspectives.

## Abbreviations

ARD: absolute risk difference
CEA: cost-effectiveness analysis
DALY: disability-adjusted life year
GBV: gender-based violence
ICER: incremental cost-effectiveness ratio
mHealth: mobile health
NECHR: National Ethics Committee for Health Research
RCT: randomized controlled trial
STI: sexually transmitted infection
