# Supplementary material for: Connecting Female Entertainment Workers in Cambodia to Health Care Services Using mHealth: Economic Evaluation of Mobile Link
Source: JMIR Form Res. 2024 Jul 25;8:e52734. doi: 10.2196/52734 (PMC11310643; doi:10.2196/52734)
Supplement: Multimedia Appendix 1 [file formative_v8i1e52734_app1.docx]

| **Sector** | **Type of Impact** | **Patient** | **Payer** | **Notes on sources of evidence** |
| --- | --- | --- | --- | --- |
| Formal healthcare sector | | | | |
| **Health** | Longevity |  |  | Not measured in the Mobile Link trial |
|  | Health-related quality-of-life effects | X | X | Measured in terms of primary and secondary trial outcomes and DALYs |
|  | Other health effects (e.g., caregiver health-related quality of life) |  |  | Not measured in the Mobile Link trial |
|  | Medical costs paid for by third-party payers |  | X | Limited to costs in the trial; see Table 1 in main text |
|  | Medical costs paid for by patients out-of-pocket |  |  | Excluded |
|  | Future related medical costs |  |  | Excluded |
|  | Future unrelated medical costs |  |  | Excluded |
| Informal healthcare sector | | | | |
| **Health** | Patient costs | X |  | Estimated using self-reported participant data on weekly income and time spent seeking care |
|  | Unpaid caregiver time costs |  |  | Excluded |
|  | Transportation costs |  |  | Excluded due to lack of data |
| Non-healthcare sectors | | | | |
| **Productivity** | Formal labor market earnings lost |  |  | Excluded |
|  | Cost of unpaid lost productivity due to illness |  |  | Excluded |
|  | Cost of uncompensated household production |  |  | Excluded |
| **Consumption** | Future consumption unrelated to health |  |  | Excluded |
| **Social services** | None |  |  |  |
| **Legal/criminal justice** | Costs associated with the justice system, police protection, judicial and legal costs, and corrections |  |  | Excluded |
| **Education** | Impact of intervention on educational achievement of population |  |  | Excluded |
| **Housing** | None |  |  |  |
| **Environment** | None |  |  |  |

The Impact Inventory allows analysts to consider all the consequences of a health intervention from various perspectives. Marks (X) indicate whether a particular impact was included in the perspective listed at the top of the column.

DALY, disability-adjusted life year.
