# Supplementary material for: Connecting Female Entertainment Workers in Cambodia to Health Care Services Using mHealth: Economic Evaluation of Mobile Link
Source: JMIR Form Res. 2024 Jul 25;8:e52734. doi: 10.2196/52734 (PMC11310643; doi:10.2196/52734)
Supplement: Multimedia Appendix 2 [file formative_v8i1e52734_app2.docx]

| **Variable** | **Intervention  (n=218)** | | **Control  (n=170)** | |
| --- | --- | --- | --- | --- |
|  | n | % | n | % |
| **Demographics** |  |  |  |  |
| Age in years (mean, SD) | 24.7 | 3.8 | 24.5 | 4.0 |
| Education in years of schooling completed (mean, SD) | 6.2 | 3.0 | 6.3 | 3.0 |
|  | | | | |
| **Marital status** |  |  |  |  |
| Currently married^*^ | 42 | 19.3 | 48 | 28.2 |
| Previously married (widowed, divorced, or separated) | 81 | 37.2 | 55 | 32.4 |
| Never married | 95 | 43.6 | 67 | 39.4 |
|  | | | | |
| **Number of dependents** (mean, SD) | 2.8 | 1.8 | 2.9 | 2.0 |
|  | | | | |
| **Province** |  |  |  |  |
| Phnom Penh | 86 | 39.4 | 51 | 30.0 |
| Battambang^†^ | 35 | 16.1 | 15 | 8.8 |
| Banteay Meanchey | 52 | 23.8 | 55 | 32.4 |
| Siem Reap | 45 | 20.6 | 49 | 28.8 |
|  | | | | |
| **Poor as a child** (Multidimensional Childhood Poverty Scale score ≥ 3) | 190 | 87.2 | 145 | 85.3 |
|  | | | | |
| **Weekly income** in USD (mean, SD) | 277.0 | 206.2 | 272.4 | 167.0 |
|  | | | | |
| **Entertainment job venue type** |  |  |  |  |
| Karaoke bar^†^ | 146 | 67.0 | 93 | 54.7 |
| Beer garden, restaurant, or café^†^ | 26 | 11.9 | 37 | 21.8 |
| Other (e.g., massage, dance club, or freelance in streets/parks) | 46 | 21.1 | 40 | 23.5 |
|  | | | | |
| **Number of months in entertainment worker industry** (mean, SD) | 30.3 | 27.0 | 28.1 | 34.9 |
|  | | | | |
| **Member of an entertainment/sex worker organization** | 16 | 7.3 | 13 | 7.6 |
|  | | | | |
| **Had sex with partner in exchange for money or gifts, last 3 months** (n=146, n=170) | 60 | 41.1 | 40 | 31.5 |
|  | | | | |
| **Type of message received** |  |  |  |  |
| Short message service (SMS) | 99 | 45.4 | 64 | 37.6 |
| Voice message (VM) | 119 | 54.6 | 106 | 62.4 |
|  |  |  |  |  |
| **Primary outcomes** |  |  |  |  |
| Tested for HIV in last six months (n=176, n=150) | 113 | 64.2 | 95 | 63.3 |
| Tested for STIs when most recently showed symptoms (n=112, n=71) | 27 | 24.1 | 19 | 26.8 |
| Uses modern contraceptive to prevent pregnancy | 68 | 31.2 | 64 | 37.6 |
| Always uses condom with non-paying partners | 43 | 71.1 | 31 | 77.5 |
| Always uses condom with paying clients (n=60, n=40) | 23 | 22.6 | 17 | 15.4 |
|  | | | | |
| **Secondary outcomes** |  |  |  |  |
| Forced drinking at work when you had no desire to drink, frequency |  |  |  |  |
| Never | 123 | 56.4 | 111 | 65.3 |
| Less than monthly | 16 | 7.3 | 13 | 7.6 |
| Monthly | 28 | 12.8 | 15 | 8.8 |
| Weekly | 51 | 23.4 | 31 | 18.2 |
| GHQ-12 score above mean (i.e., higher severity of psychiatric condition) | 11 | 42.3 | 25 | 33.8 |
| Gender-Based Violence Acceptance Scale Score, range = 0-16 (mean, SD) (n=26, n=74) | 4.4 | 3.7 | 4.5 | 3.2 |
| Believes you cannot do anything if you or someone you know experiences physical or sexual abuse | 60 | 27.5 | 47 | 27.6 |
|  | | | | |
| **Outreach worker contact only**, last six months | 20 | 9.2 | 13 | 7.7 |
|  | | | | |
| **Number of times contacting outreach worker**, last six months |  |  |  |  |
| Never | 20 | 46.5 | 21 | 58.3 |
| Once | 11 | 25.6 | 6 | 16.7 |
| Two to four times | 11 | 25.6 | 8 | 22.2 |
| Five or more times | 1 | 2.3 | 1 | 2.8 |
|  | | | | |
| **Received escorted referral** |  |  |  |  |
| For HIV/STI (n=23, n=23) | 7 | 30.4 | 4 | 17.4 |
| For vaginal Health (n=23, n=23) | 14 | 60.9 | 16 | 69.6 |

^*^Indicates a significant difference between intervention and control arms at baseline in cluster-adjusted test of association by venue (p<0.05).

^†^Indicates a significant difference between intervention and control arms at baseline in crude test of association (p<0.05).

GHQ-12, General Health Questionnaire; HIV, human immunodeficiency virus; SD, standard deviation; STI, sexually transmitted infection.
