# Supplementary material for: Connecting Female Entertainment Workers in Cambodia to Health Care Services Using mHealth: Economic Evaluation of Mobile Link
Source: JMIR Form Res. 2024 Jul 25;8:e52734. doi: 10.2196/52734 (PMC11310643; doi:10.2196/52734)
Supplement: Multimedia Appendix 3 [file formative_v8i1e52734_app3.docx]

| **Primary outcome** | **OR** | **95% CI** | | **AOR** | **95% CI** | |
| --- | --- | --- | --- | --- | --- | --- |
| Tested for HIV, last six months (1=Yes, 0=No) (n=887) | 0.450 | 0.179 | 1.130 | 0.404 | 0.156 | 1.043 |
| Tested for STIs, most recent symptoms (1=Yes, No=0) (n=394) | 1.355 | 0.245 | 7.499 | 1.200 | 0.205 | 7.004 |
| Uses modern contraceptive to prevent pregnancy (1=Yes, No=0) (n=989) | 1.061 | 0.373 | 3.021 | 0.985 | 0.349 | 2.774 |
| Always uses condom with non-paying partners (1=Yes, No=0) (n=586) | 0.264* | 0.084 | 0.831 | 0.496 | 0.156 | 1.578 |
| Always uses condom with paying clients (1=Yes, No=0) (n=242) | 1.767 | 0.137 | 22.742 | 1.170 | 0.078 | 17.635 |

Adjusted models included venue type, province, cohabitation, age, education, and outreach worker contact.

AOR, adjusted odds ratio; CI, confidence interval; HIV, human immunodeficiency virus; OR, odds ratio; STI, sexually transmitted infection.
