# Supplementary material for: Connecting Female Entertainment Workers in Cambodia to Health Care Services Using mHealth: Economic Evaluation of Mobile Link
Source: JMIR Form Res. 2024 Jul 25;8:e52734. doi: 10.2196/52734 (PMC11310643; doi:10.2196/52734)
Supplement: Multimedia Appendix 4 [file formative_v8i1e52734_app4.docx]

| **Secondary outcome** | **OR** | **95%CI** | | **AOR** | **95%CI** | |
| --- | --- | --- | --- | --- | --- | --- |
| Outreach worker contact, last six months (1=Yes, 0=No) | 3.306 | 1.058 | 10.332 | 2.816 | 0.927 | 8.554 |
| Escorted referral, last six months (1=Yes, 0=No) | 9.509 | 2.057 | 43.949 | 8.150 | 1.650 | 40.250 |
| Forced drinking at work, last three months (1=Never, 0=Ever) | 4.280 | 1.720 | 10.648 | 3.945 | 1.622 | 9.597 |
| Believes you can do something if experience abuse (1=Yes, 0=No) | 0.651 | 0.267 | 1.588 | 0.677 | 0.284 | 1.615 |
| Gender-based violence (1=High/moderate, 0=Low) (n=701) | 0.807 | 0.251 | 2.590 | 0.861 | 0.276 | 2.679 |

^*^Adjusted models include venue type, province, cohabitation, age, and education.
AOR, adjusted odds ratio; CI, Confidence interval; OR, odds ratio.
