# Supplementary material for: Connecting Female Entertainment Workers in Cambodia to Health Care Services Using mHealth: Economic Evaluation of Mobile Link
Source: JMIR Form Res. 2024 Jul 25;8:e52734. doi: 10.2196/52734 (PMC11310643; doi:10.2196/52734)
Supplement: Multimedia Appendix 5 [file formative_v8i1e52734_app5.docx]

**Multimedia Appendix 5. Calculating disability weights for each Mobile Link trial outcome**

The process of estimating disability weights for each outcome are explained in detail below.

**HIV testing**

The benefit of HIV testing is twofold: reducing the anxiety associated with not knowing one’s status and improving access to HIV care following a positive screen.

We took disability weights for uncomplicated HIV (0.012) and early HIV (0.159) from the 2019 Global Burden of Diseases (GBD) study (Multimedia Appendix 6) [25]. To reflect the fact that high HIV risk poses psychological distress to FEWs [30], we assumed that the disability associated with early detection of HIV is the difference between early HIV and uncomplicated HIV. We then multiplied the difference (0.147) by the adjusted prevalence of HIV among FEWs in Cambodia (3.2%) [31] and by the probability of HIV care and antiretroviral therapy (83%) [32]. The product (0.004) is the final disability weight for each FEW with HIV who is tested and treated.

**STI testing**

We followed a similar approach used in HIV testing to estimate the disability weight for sexually transmitted infection (STI) testing.

We multiplied the disability weight for mild STI from the GBD (0.006) by the prevalence of STI among FEWs and the probability of STI treatment. Based on data from the Mobile Link trial, about 34% of tested FEWs were positive for an STI, and 80% of them were treated. Together, this translates to a 27% chance of STI treatment following an STI test. The final disability weight for every FEW with an STI who is tested and treated is 0.002.

**Modern contraceptive use**

We used the 5^th^ version of the Impact 2 model developed my Marie Stopes International to estimate the average disability weight associated with modern contraceptive use for pregnancy prevention. Impact 2, which has been described in detail in previous publications [26, 27], is an Excel-based model designed to estimate the impact of reproductive services provided by an organization in one or more countries. The model is pre-populated with country-specific data from Demographic and Health Surveys, UN Population, World Health Organization, and GBD, among others. Data on the effectiveness of modern, long-acting, and permanent contraceptive methods in preventing pregnancy is based on peer-reviewed literature. Impact 2 has been used in prior economic evaluations in Cambodia [28].

In Impact 2, the total disability adjusted life years (DALYs) associated with modern contraceptive use is a function of averted unintended pregnancies, safe (i.e., facility-based) and unsafe abortions, and maternal mortality. (Impact 2 also estimates the DALYs from under-five mortality averted, but we excluded this from the analysis.) The Table shows the estimated DALYs averted by each additional use of modern contraceptive methods from Impact 2 for Cambodia. We took the average DALYs across all contraceptive methods to determine the final disability weight used in the CEA. In practice, most FEWs use male condoms for pregnancy prevention, followed by birth control pills, and injectable contraception [33].

**Table.** Disability-adjusted life years (DALYs) averted per family planning service or commodity used

| **Contraceptive method** | **DALYs averted** |
| --- | --- |
| Female sterilization | 0.05949 |
| Implant (5 year) | 0.03490 |
| Implant (4 year) | 0.03065 |
| Implants (3 year) | 0.02526 |
| IUD (10 year) | 0.03629 |
| IUD (5 year) | 0.03127 |
| IUS (5 year) | 0.03127 |
| IUS (3 year) | 0.02382 |
| Condoms (free) | 0.00005 |
| Condoms (paid) | 0.00005 |
| Female condoms (free) | 0.00005 |
| Female condoms (paid) | 0.00005 |
| Pills (cycles) | 0.00057 |
| Diaphragm | 0.00666 |
| Foam tablets | 0.00005 |
| Injectables (1 month) | 0.00067 |
| Injectables (2 month) | 0.00134 |
| Injectables (3 month) | 0.00201 |
| Vaginal ring | 0.00057 |
| Contraceptive Patch | 0.00057 |
| Average | 0.014 |

IUD, intrauterine device; IUS, intrauterine system.

**Forced drinking at work**

We used the disability weight for very mild alcohol use disorder (AUD) in the GBD. While forced drinking at work may not be equivalent to very mild AUD, qualitatively, these two states have similar characteristics. Data from the Mobile Link trial show a positive association between the number of standard drinks on a typical day with the experience of being forced to drink at work [34]. Additionally, qualitative studies have revealed that FEWs who drink while working find it harder to perform certain tasks and activities, such as ensuring that their clients are wearing protection [1, 29, 35], which matches the description of very mild AUD in the GBD (Multimedia Appendix 6).

**Gender-based violence**

We estimated the disability weight for gender-based violence using a similar approach used in the GBD to estimate sexual violence [36]. We assumed that FEWs who experience high gender-based violence face both physical and mental harms, which is consistent with the items listed in the gender-based violence section of the questionnaire in the Mobile Link trial and studies on violence on women, intimate partners, and sex workers [37-41]. The disability weight associated with physical harm were estimated by taking the average disability weight across five, increasingly severe, short-term head, neck, and musculoskeletal injuries, from muscle and tendon injuries (e.g., sprains) to pelvic fractures (Multimedia Appendix 6). The disability weight associated with mental harm from GBV were based on the average disability weight for mild anxiety disorder and mild major depressive disorder. The total disability weight associated with GBV is the sum of the average disability weight of physical and mental harm.
