# Supplementary material for: Connecting Female Entertainment Workers in Cambodia to Health Care Services Using mHealth: Economic Evaluation of Mobile Link
Source: JMIR Form Res. 2024 Jul 25;8:e52734. doi: 10.2196/52734 (PMC11310643; doi:10.2196/52734)
Supplement: Multimedia Appendix 6 [file formative_v8i1e52734_app6.docx]

| **Outcome in Mobile Link trial** | **Matched health state from GBD** | **GBD health state description** | **Mean disability weight (range)** |
| --- | --- | --- | --- |
| HIV testing | Generic uncomplicated disease: anxiety about diagnosis | Has a disease diagnosis that causes some worry but minimal interference with daily activities. | 0.012 (0.006-0.023) |
| HIV testing | Early HIV with severe anemia | Anemia, severe; Generic uncomplicated disease anxiety | 0.159 (0.109-0.22) |
| STI testing | Infectious disease, acute episode, mild^*^ | Has a low fever and mild discomfort, but no difficulty with daily activities | 0.006 (0.002-0.012) |
| Forced drinking at work | Alcohol use disorder, very mild | Drinks a lot of alcohol and sometimes has difficulty controlling the urge to drink. While intoxicated, the person has difficulty performing daily activities. | 0.123 (0.082-0.177) |
| Gender-based violence - physical | Other injuries of muscle and tendon (includes sprains, strains and dislocations other than shoulder, knee, hip) | Has a strained muscle that causes pain and swelling | 0.008 (0.003-0.015) |
| Gender-based violence - physical | Fracture of patella, tibia or fibula or ankle (short term, with or without treatment) | Has a broken shin bone, which causes severe pain, swelling, and difficulty walking | 0.05 (0.032-0.075) |
| Gender-based violence - physical | Fracture of face bone (short or long term, with or without treatment) | Has a broken cheek bone, broken nose, and chipped teeth, with swelling and severe pain | 0.067 (0.044-0.097) |
| Gender-based violence - physical | Concussion | Has headaches, dizziness, nausea and difficulty concentrating | 0.214 (0.141-0.297) |
| Gender-based violence - physical | Fracture of pelvis (short term) | Has a broken pelvis bone, with swelling and bruising. The person has severe pain, and cannot walk or do daily activities | 0.279 (0.188-0.384) |
| Gender-based violence - mental | Anxiety disorders, mild | Feels mildly anxious and worried, which makes it slightly difficult to concentrate, remember things, and sleep. The person tires easily but is able to perform daily activities. | 0.03 (0.018-0.046) |
| Gender-based violence - mental | Major depressive disorder, mild episode | Feels persistent sadness and has lost interest in usual activities. The person sometimes sleeps badly, feels tired, or has trouble concentrating but still manages to function in daily life with extra effort. | 0.145 (0.099-0.209) |

^*^Same health state description and disability weight for mild chlamydia, gonorrhea, and syphilis infections.

GBD, Global Burden of Diseases study.
