# Supplementary material for: Connecting Female Entertainment Workers in Cambodia to Health Care Services Using mHealth: Economic Evaluation of Mobile Link
Source: JMIR Form Res. 2024 Jul 25;8:e52734. doi: 10.2196/52734 (PMC11310643; doi:10.2196/52734)
Supplement: Multimedia Appendix 7 [file formative_v8i1e52734_app7.docx]

|  | **Control group (n=900)** | **Intervention group (n=822)** | **Incremental cost of Mobile Link** |
| --- | --- | --- | --- |
| Total costs (payer perspective) | 207,155 | 352,382 | 145,226 |
| Cost per participant (payer perspective) | 230 | 429 | 199 |
| Cost per participant (combined payer and patient perspective) | 227 | 422 | 195 |

Sample sizes include all participants recruited during the trial, including those who eventually dropped out. All costs are in US$.
